# Microbial metacommunity dynamics associated with the cyclopoid copepod *Mesocyclops hyalinus* (Rehberg, 1880) in freshwater mesocosm using low-cost organic media

**DOI:** 10.3389/fsysb.2026.1845217

**Published:** 2026-08-11

**Authors:** Rida Riyaz, Sukham Munilkumar, Na-i-sabet Dohtdong, Paramita Banerjee Sawant, Perumal Santhanam, Madhuri Pathak, S. P. Shukla, Pradeep Kumar Singh, Kenyum Lollen, Pranab Gogoi

**Affiliations:** 1 Division of Aquaculture, Indian Council of Agricultural Research-Central Institute of Fisheries Education (ICAR-CIFE), Versova, Mumbai, India; 2 Department of Marine Science, Bharathidasan University, Tiruchirappalli, Tamil Nadu, India; 3 Krishi Vigyan Kendra Mungeli, Indira Gandhi Krishi Vishwavidyalaya, Raipur, Chhattisgargh, India; 4 Indian Council of Agricultural Research-Central Inland Fisheries Research Institute, Barrackpore, West Bengal, India

**Keywords:** 16S rRNA sequencing, copepods, mesocosm, *Mesocyclops hyalinus*, organic manure

## Abstract

This mesocosm study evaluated the impact of various organic manure treatments on the growth performance of the copepod *M. hyalinus* and its associated microbial communities. Laboratory reared copepod, *M. hyalinus* was fed with six different types of filtrate of organic manure comprising of CM (cow manure), PM (poultry manure) and combinations CMC* (Cow dung, groundnut cake, mustard oil cake, rice bran, fish waste and single super phosphate), PMC** (Poultry manure, groundnut cake, mustard oil cake, rice bran, fish waste and single super phosphate), PMB*** (PMC + *Bacillus clausii*), CMB**** (CMC + *B. clausii*). Results indicated that CMC, PMC, and CMB at a concentration of 8.00 mL/L produced the highest population densities. CMC achieved a peak of 650.00 ± 11.55 Individuals/L by Day 6, while PMC and CMB peaked later on Day 9 (463.33 ± 6.67 Individuals/L) and Day 15 (563.33 ± 6.67 Individuals/L), respectively. 16S rRNA amplicon sequencing analysis of the high performing media (CMC and PMC) identified *Actinobacteriota*, *Proteobacteria*, *Firmicutes*, and *Bacteroidota* as the dominant bacterial phyla. In CMC, *Actinobacteriota* (54%) and *Proteobacteria* (20%–47%) predominated at peak growth. In contrast, PMC was dominated by *Firmicutes* (20%–39%) and *Campylobacterota* (26%–28%). These findings suggest that manure composition significantly dictates microbial structure and subsequent copepod productivity; cow manure based media promote rapid early growth, whereas poultry manure supports sustained population stability. The identified bacterial consortia offer a framework for developing targeted bioinoculants to optimize *Mesocyclops hyalinus* culture systems. By harnessing these microbiome driven insights, producers can enhance copepod yields and population stability. This research provides a scalable, sustainable strategy for mass producing live feed, addressing a critical bottleneck in global freshwater aquaculture.

## Highlights

- The population density of *Mesocyclops hyalinus* increased when fed the filtrate of an organic manure combination.
- CMC treatment supported the highest copepod density (650 ± 12 ind/L) by Day 6.
- Probiotic addition with *Bacillus clausii* did not improve copepod population density when added to poultry manure based combination. The addition of *B. clausii* to the cow manure based extract increased copepod density significantly only by Day 15, suggesting a delayed probiotic effect.
- 16S rRNA analysis revealed distinct microbial communities between cow and poultry manure treatments.
- Microbial succession towards probiotic dominated communities appears to have facilitated *M. hyalinus* survival and reproductive success.
- Optimised organic manure blends can enhance low-cost livefeed production in aquaculture.

## 1 Introduction

Aquaculture is one of the fastest growing food production sectors, relying on the availability of nutritionally balanced live feeds for successful larval rearing (Das et al., 2012; Vidhya et al., 2014). Early larval mortality continues to be a major bottleneck in both marine (Turner et al., 1985) and freshwater species (Jana and Jana, 2003), largely due to nutritional imbalances, poor water quality, diseases, and predation (Frimpong and LochMann, 2005). Copepods are recognized as nutritionally most valuable livefeed in aquaculture, particularly during early larval stages. Their suitable size range, enhanced nutritional quality, digestibility, and high PUFA makes them superior to traditional livefeeds like Artemia and Rotifers (Melianawati et al., 2015; Karlsen et al., 2015). In extensive and semi-intensive aquaculture systems, naturally occurring copepods significantly enhance larval survival, growth performance and overall pond productivity. Consequently, management practices such as organic fertilization is a widely adopted practice for promoting copepod productivity through natural food web development. The application of organic manures such as cow dung, poultry manure, plant based residues supply organic matter and nutrients that are gradually released through microbial decomposition, thereby promoting growth of phytoplankton, bacterial biomass and detrital pathways (Shigang, 1989). These nutrient driven cascades ultimately support zooplankton growth, including copepods, which form a critical link between primary producers and higher trophic levels in ponds (Shigang, 1989; Rajanna et al., 2021). The effectiveness of organic fertilizers in enhancing copepod production is largely mediated by microbial communities inhabiting the sediment water interface. The microbial assemblage therefore constitutes a direct or supplementary food source for copepods (Tang, 2005). Organic fertilizers can enhance microbial biomass under optimal carbon-to-nitrogen (C/N) ratios; improve water quality and nitrogen assimilation (Azam et al., 1983; Avnimelech, 2003; Schmidt et al., 2016; De Corte et al., 2018). The bacterial biomass also plays key role in breaking down complex organic matter, releasing essential nutrients, synthesizing vital vitamins and cofactors that enhance metabolic efficiency, reproduction, and immunity of copepods (Gorokhova et al., 2021). The dynamic host-microbe interactions contribute to population stability and energy balance within aquatic ecosystems (Schmidt et al., 2016). Despite their predominant role, the microbial mechanisms underlying fertilizer induced changes in copepod population dynamics remain insufficiently understood. Studying these complex interactions directly in aquaculture ponds is often constrained by environmental heterogeneity and limited experimental control. Mesocosm experiments provide a robust intermediate approach between laboratory mesocosm and field scale studies by simulating key ecological processes of a fertilized pond system under controlled and replicable conditions (Wang et al., 2021). Therefore, the present study employed small scale freshwater mesocosm to evaluate the effect of six different organic fertilizers on growth and population dynamics of copepod *M. hyalinus.*

*Mesocyclops hyalinus* is a medium sized cyclopoid copepod (0.7–1.1 mm) and therefore falls within the optimal prey size range required by many freshwater fish larvae, making it an efficient live feed. Also, the specie is widely distributed across Europe, Asia, Africa and Australia therefore, reflecting its broad ecological tolerance and environmental plasticity (Ueda and Reid, 2003). To explore the microbial interactions in *M. hyalinus*, we employed high throughput sequencing to track changes in population growth alongside shifts in bacterial diversity and community structure across different developmental stages. Integrating a 16S rRNA study with mesocosm based experiment offers a powerful framework to elucidate microbiome mediated pathways linking organic fertilization to copepod growth and population dynamics. Consequently the present study aimed to: (1) determine the optimal organic manure filtrate for the mass production of *M. hyalinus*, and (2) elucidate the microbial successional patterns that support copepod productivity.

## 2 Materials and methods

### 2.1 Experimental setup

**FIGURE 1:**
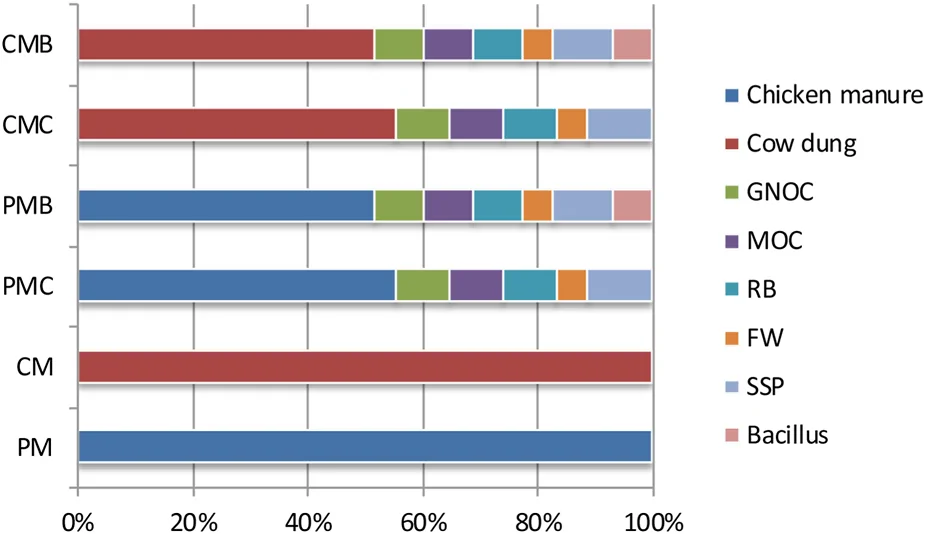
Organic manure treatment combinations (gm/L). PM: Poultry manure, CM: Cow manure, PMC: Poultry manure combination, PMB: Poultry manure combination + *Bacillus clausii* CMC: Cow manure combination, CMB: Cow manure combination + *Bacillus clausii*, GNOC: Groundnut oil cake, MOC: Mustard oil cake, RB: Rice bran, FW: Fish waste, SSP: Single super phosphate.

The experiment was conducted using freshwater mesocosm to simulate natural conditions under laboratory settings. The culture of *M. hyalinus* was conducted in the wet lab facility of ICAR- Central Institute of Fisheries Education, Mumbai, India. The experiment was conducted in triplicate using six different diets given in Figure 1 with each treatment set up in 5 L HDPE tank containing a layer of sediment overlaid with filtered source water. Sediment and source water were obtained from the aquaculture pond facilities at the ICAR-CIFE wet lab. Water was filtered using filter-bag (1µ) to minimize the influence of macro-particulates and large planktonic organisms. The soil was spread in thin layers and exposed to direct solar radiation for thermal inactivation of soil borne pathogens and pests (An-nori et al., 2022). The diets regime consisted of organic manure filtrates prepared following the protocol described by Ogello et al. (2018) and Jana and Chakrabarti (1993). The fermentation of organic manure by adding *B. clausii* was done according to Suminto et al. (2018). The inclusion of sediment facilitated nutrient mineralization and microbial activity, whereas organic manures served as nutrient sources. Before experiment, the copepods from the pre-culture were harvested and transferred to the new culture water for a 24 h gut evacuation to avoid the effects of residential diet (Pan et al., 2018). The copepods were fed on six different regimes (treatments), at a concentration of 8.00 mL/L per day. The experiment lasted 15 days.

Water samples (30 mL) from indoor culture were collected concurrently on the initial and peak population days of the copepod culture, specifically on days 1 and 6 for the cow manure combination and days 1 and 9 for the poultry manure combination. The culture medium was then filtered using a 40 µm mesh to remove macro-particles before further analysis. To evaluate macro-level shifts in microbial community structures, an exploratory, pilot-scale study design was implemented using n = 2 biological replicates per treatment group at each designated timepoint, resulting in a total dataset of N = 8 samples. While microbial amplicon data inherently exhibit pronounced biological and inter-individual variability, this sample allocation is consistent with established frameworks for baseline pilot-scale sequencing surveys.

To mitigate the statistical impacts of biological variance and ensure a robust downstream ecological characterization, a high coverage sequencing strategy was prioritized. This approach yielded extensive read depth across all processed cohorts, with raw sequences ranging between 417,588 and 852,188 reads per individual sample. This deep sequencing coverage was leveraged to minimize technical artifact noise, provide stable estimations of community alpha and beta diversity, and secure comprehensive representation of the low-abundance rare biosphere.

### 2.2 Population growth experiment

From the pre-culture, 50 Individual/L were inoculated into 18 5 L HDPE tanks (6 culture media × 3 replicates), each containing 8.00 mL of manure extract per litre. The cultures were maintained under optimal physico-chemical conditions. Population enumeration was conducted on days 3, 6, 9, 12, and 15 (Magoz et al., 2021). Every 3 days, 100 mL of water was randomly sampled from each triplicate to estimate the population growth of copepods, recorded as an increase in number (Individual/L). 100 mL of water sampled for population counts was immediately replaced with fresh filtered source water containing the corresponding treatment filtrate concentration to maintain constant volume.

Based on the recorded data, the intrinsic rate of natural increase, doubling time (in Days), and population growth were calculated following the equation described by James and Dias (1984).

r = Ln (Nt)-Ln (No)/t (James and Dias, 1984)
Td = 0.6931/r (James and Dias, 1984)
Nt = No. e^rt^ (James and Dias, 1984)

Where,

r = the intrinsic rate of natural increase.
Nt and No. = final and initial populations.
Td = Doubling time of the population in days.

### 2.3 DNA extraction and DNA QC

Culture water samples were centrifuged at 4,000 × g for 30 min, and the resulting supernatant was discarded. The pellet was resuspended in CD1 lysis buffer and incubated at 65 °C for 10 min. DNA extraction was subsequently performed using the QIAamp PowerFecal Pro DNA Kit (Cat No./ID: 51804, QIAGEN GmbH), following the manufacturer’s instructions. The extracted DNA was quantified using a NanoDrop One spectrophotometer and Qubit fluorometer, with elution buffer and standards serving as controls, respectively. To assess DNA integrity, an aliquot of the extracted DNA was subjected to electrophoresis on a 0.8% agarose gel prepared in 1× TAE buffer, alongside a 1 kb DNA ladder. The gel was stained with SYBR Safe dye and visualized under UV illumination.

### 2.4 16S rRNA V3-V4 metagenomic library preparation

Metagenomic libraries were prepared following the 16S Metagenomic Library Preparation Protocol from Illumina Inc. The gene-specific primers used in this protocol target the V3-V4 region of the 16S rRNA gene, as described by Klindworth et al. (2013), and represent an optimal bacterial primer pair. Illumina adapter overhang nucleotide sequences were incorporated into the gene-specific primers. The complete primer sequences, provided in IUPAC nucleotide nomenclature, are as follows:

16S amplicon PCR forward primer: 5′-TCGTCGGCAGCGTCAGATGTGTATAAGAGACAGCCTACGGGNGGCWGCAG-3'.

16S amplicon PCR reverse primer: 5′GTCTCGTGGGCTCGGAGATGTGTATAAGAGACAGGACTACHVGGGTATCTAATCC-3'.

For library construction, 12.5 ng of extracted DNA from each sample was used as input for 16S V3-V4 PCR amplification with the respective primers. The amplified PCR products were purified using bead-based purification and subjected to a second round of PCR for the addition of dual indices and Illumina adapters, as per the standard Illumina workflow. Purified libraries were quantified using a Qubit fluorometer. To assess the fragment size distribution and average library size, appropriate dilutions were analyzed on a D1000 ScreenTape using an Agilent TapeStation system.

### 2.5 Bioinformatics analysis

Raw paired end reads were imported into the QIIME2 ecosystem (v2022.10) using a manifest file format. Sequence quality was initially assessed, followed by denoising via the DADA2 plugin (denoise-paired). To ensure high-quality data and prevent the false-positive detection of chimeras often caused by primer degeneracy primers were removed using a 17 bp left trim and a 21 bp right trim. Based on quality score profiles, sequences were truncated at 290 bp for both forward and reverse reads. This process yielded 2,619 exact Amplicon Sequence Variants (ASVs) with a mean length of 273 bp. To facilitate robust taxonomic assignment within a phylogenetically consistent framework, we utilized the Greengenes2 (v2022.10) database. While DADA2 inherently produces ASVs, we employed the Greengenes2 “non-v4-16s” workflow to perform closed-reference clustering via the q2-vsearch plugin. This step was taken to map our experimental sequences against the Greengenes2 backbone of full-length 16S rRNA sequences (2022.10. backbone.full-length.fna.qza). This approach leverages the massive, whole-genome-based phylogeny of Greengenes2, resulting in a refined set of 939 high-confidence features (mean length 313 bp) that are compatible with the database’s integrated taxonomic and phylogenetic tree. Taxonomy was assigned to the resulting features using the taxonomy-from-table plugin, referencing the 2022.10. taxonomy.asv.nwk.qza file. The final feature table, taxonomy, and associated metadata were then integrated into a phyloseq object for downstream ecological and statistical analyses.

To ensure absolute methodological clarity, we explicitly emphasize that the 939 post-mapping features were retained at 100% exact sequence resolution as distinct ASVs and were not collapsed, binned, or clustered into traditional *de novo* Operational Taxonomic Units (OTUs). Within the Greengenes2 framework, the ‘closed-reference’ mapping step functions strictly as a high-stringency sequence-matching and quality-control filter. Instead of clustering sequences into arbitrary similarity thresholds, individual ASVs are matched against a fixed, genomic-backed backbone tree. Features that do not map are discarded—explaining the reduction from 2,619 to 939 features while those that pass are preserved with their original sequence fidelity intact. Consequently, this approach does not compromise ecological interpretation. By maintaining exact ASV resolution while assigning taxonomy via the integrated phylogenetic tree, we preserve fine-scale biological variation and secure the high taxonomic resolution necessary for accurate downstream diversity and community abundance analyses.

### 2.6 Statistical analysis

#### 2.6.1 Statistical analysis

Data were analyzed using SPSS version 22 and R. One-way ANOVA followed by Duncan’s multiple range test was used to compare copepod population density among treatments at *p* < 0.05. Taxonomic abundance was analyzed using the Mann–Whitney U test for independent biological replicates, with one sample taken from each replicate per treatment. Significance was shown at *p* < 0.05, *p* < 0.01, and *p* < 0.001. No formal multiple-testing correction was applied, and the taxonomic results were interpreted as exploratory. Beta diversity was assessed using Bray–Curtis dissimilarity and visualized by PCoA; PERMANOVA was not performed because the sample size per treatment was only two.

## 3 Results

### 3.1 Population growth of *Mesocyclops hyalinus*

**FIGURE 2:**
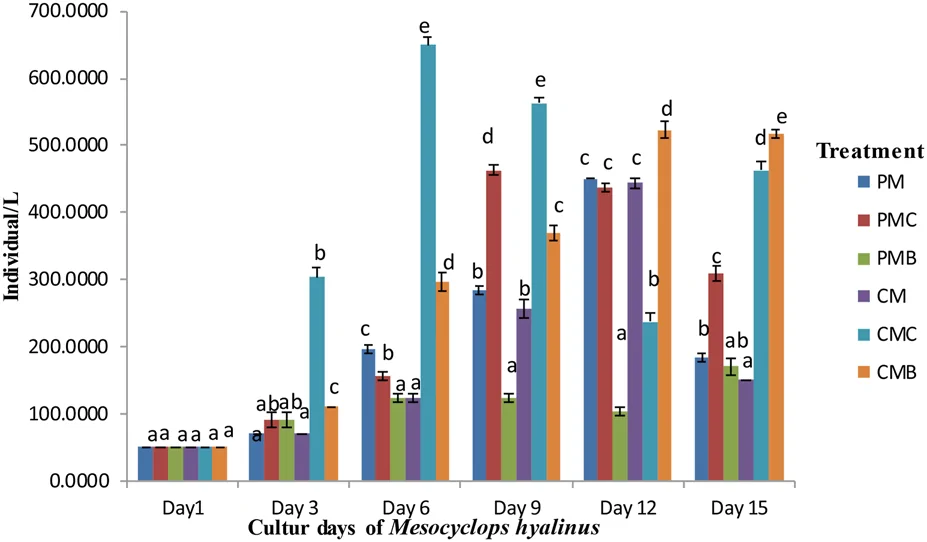
Effect of organic manure diets on population density of *Mesocyclops hyalinus* (Individual/L).

The growth performance of *M. hyalinus* varied significantly across different organic media diets (Table 1; Figure 2). Manure combinations had a significant effect on population density (*p* < 0.05). Among all treatments, the CMC group supported the highest copepod densities from Day 3 to Day 9, reaching a peak on Day 6. In poultry based treatments, the highest density was recorded in PMC on Day 9, though it was significantly lower than CMC on the same day. Intrinsic growth rate (Table 2) (r) was significantly higher in the CMC group from Day 3 to Day 9 (*p* < 0.05), with the highest value observed on Day 3. In poultry based treatments, the highest *r* was on Day 9, which was however, significantly lower than that of CMC on the same day (Table 2). Doubling time (Td) (Table 3) was significantly lower in the CMC group from Day 3 to Day 9, indicating faster reproduction (*p* < 0.05), with the shortest doubling time recorded on Day 3. In poultry based treatments the lowest doubling time (Td) was reported on Day 9 in PMC, which was however, significantly higher than that of CMC on the same day (*p* < 0.05).

**TABLE 1:** Effect of organic manure diets on population density of *Mesocyclops hyalinus* (Individual/L).

Treatment	Day 1	Day 3	Day 6	Day 9	Day 12	Day 15
PM	50.00	70.00 ± 0.00^a^	196.67 ± 6.67^c^	283.33 ± 6.67^b^	450.00 ± 0.00^c^	183.33 ± 6.67^b^
PMC	50.00	90.00 ± 11.55^ab^	156.67 ± 6.67^b^	463.33 ± 6.67^d^	436.67 ± 6.67^c^	310.00 ± 11.55^c^
PMB	50.00	90.00 ± 11.55^ab^	123.33 ± 6.67^a^	123.33 ± 6.67^a^	103.33 ± 6.67^a^	170.00 ± 11.55^ab^
CM	50.00	70.00 ± 0^a^	123.33 ± 6.67^a^	256.67 ± 13.33^b^	443.33 ± 6.67^c^	150.00 ± 0.00^a^
CMC	50.00	303.33 ± 13.33^c^	650.00 ± 11.55^e^	563.33 ± 6.67^e^	236.67 ± 13.33^b^	463.33 ± 13.33^d^
CMB	50.00	110.00 ± 0^b^	296.67 ± 13.33^d^	370.00 ± 11.55^c^	523.33 ± 13.33^d^	516.67 ± 6.67^e^

Mean values in the same column with different superscript (a,b,c,d,e) differ significantly (p < 0.05).

**TABLE 2:** Intrinsic growth rate of *Mesocyclops hyalinus*.

Treatment	Day 3	Day 6	Day 9	Day 12	Day 15
PM	0.11 ± 0.00^a^	0.23 ± 0.01^c^	0.20 ± 0.00^c^	0.18 ± 0.00^c^	0.09 ± 0.00^b^
PMC	0.19 ± 0.04^ab^	0.19 ± 0.01^b^	0.24 ± 0.00^e^	0.18 ± 0.00^c^	0.12 ± 0.00^c^
PMB	0.19 ± 0.04^ab^	0.15 ± 0.01^a^	0.10 ± 0.01^a^	0.06 ± 0.01^a^	0.08 ± 0.01^ab^
CM	0.11 ± 0.00^a^	0.15 ± 0.01^a^	0.18 ± 0.01^b^	0.18 ± 0.00^c^	0.07 ± 0.00^a^
CMC	0.60 ± 0.01^c^	0.43 ± 0.00^e^	0.27 ± 0.00^d^	0.13 ± 0.00^b^	0.15 ± 0.00^d^
CMB	0.26 ± 0.00^b^	0.29 ± 0.01^d^	0.22 ± 0.00^d^	0.19 ± 0.00^d^	0.15 ± 0.00^d^

Mean values in the same column with different superscript (a,b,c,d,e) differ significantly (p < 0.05).

**TABLE 3:** Doubling time of *Mesocyclops hyalinus* in diferent organic media.

Treatment	Day 3	Day 6	Day 9	Day 12	Day 15
PM	6.2 ± 0.0^c^	3.0 ± 0.1^c^	3.6 ± 0.1^b^	3.8 ± 0.0^ab^	8.0 ± 0.2^c^
PMC	4.1 ± 1.1^b^	3.7 ± 0.2^d^	2.8 ± 0.0^a^	3.8 ± 0.0^ab^	5.7 ± 0.1^b^
PMB	4.1 ± 1.1^b^	4.7 ± 0.5^e^	7.0 ± 0.8^c^	11.8 ± 1.2^c^	8.6 ± 0.5^c^
CM	6.2 ± 0.0^c^	4.7 ± 0.5^e^	3.8 ± 0.2^b^	3.8 ± 0.0^ab^	9.5 ± 0.0^d^
CMC	1.2 ± 0.0^a^	1.6 ± 0.0^a^	2.6 ± 0.0^a^	5.4 ± 0.2^b^	4.7 ± 0.1^a^
CMB	2.6 ± 0.0^ab^	2.3 ± 0.1^b^	3.1 ± 0.1^ab^	3.5 ± 0.0^a^	4.5 ± 0.0^a^

Mean values in the same column with different superscript (a,b,c,d,e) differ significantly (p < 0.05).

### 3.2 Alpha diversity index

**FIGURE 3:**
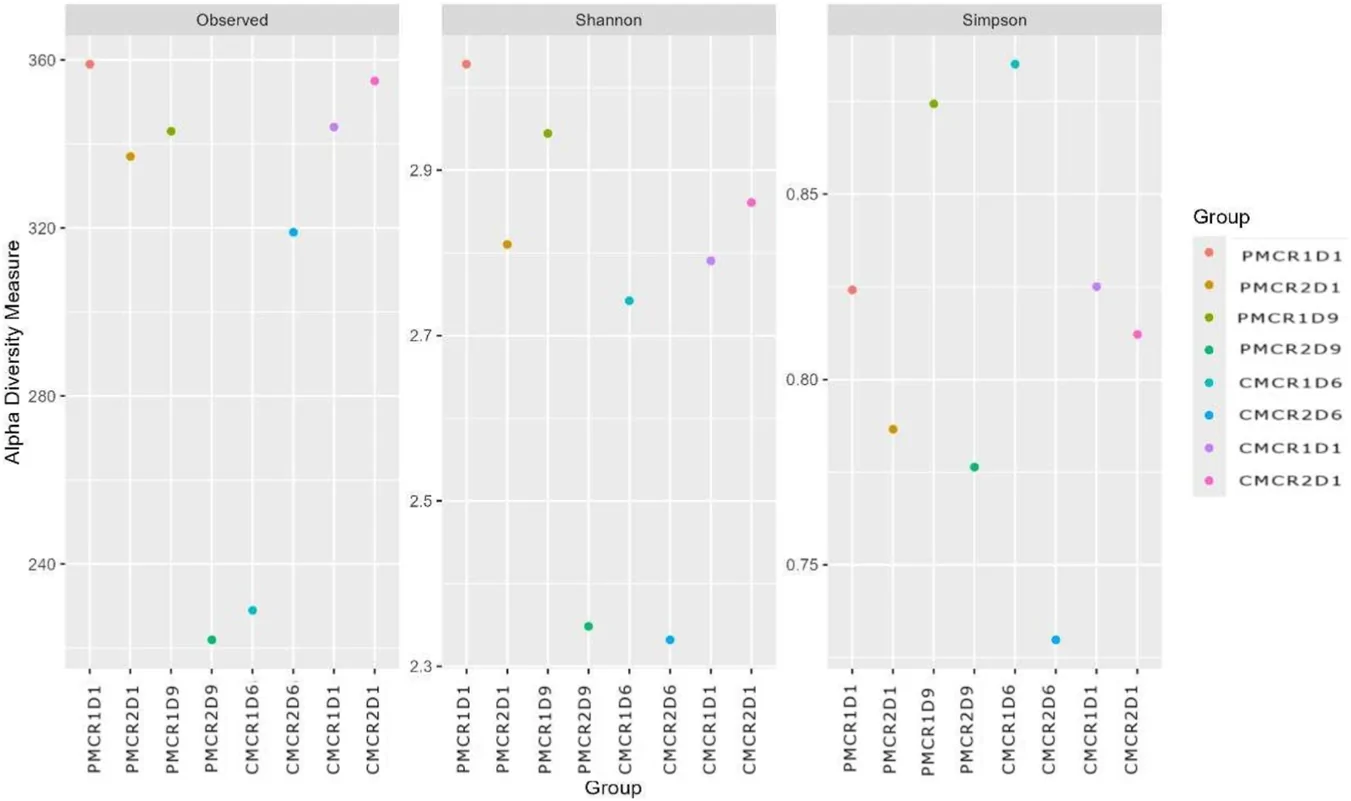
Box plot shows the alpha-diversity indices comparison between poultry manure combination and cow manure combination.

Microbial community richness and evenness were evaluated using Observed ASVs, Shannon, and Simpson indices (Table 4; Figure 3). Initially, both manure types exhibited similar richness, with Day 1 Observed ASVs ranging from 337 to 359 in poultry manure-based extracts (PMC) and 344–355 in cow manure-based extracts (CMC). A temporal decline in richness was observed in both treatments. In PMC, Observed ASVs decreased to 222–343 by Day 9, while in CMC, richness decreased to 229–319 by Day 6. Shannon diversity indices followed a similar downward trend, decreasing from 2.80 to 3.02 to 2.34–2.94 in PMC, and from 2.79 to 2.86 to 2.33–2.74 in CMC. In contrast, the Simpson index remained relatively stable across the culture period for both manure types, ranging from 0.77 to 0.87 in PMC and 0.72–0.88 in CMC.

**TABLE 4:** Three alpha diversity indices.

Sample id	Observed	Shannon	Simpson
PMCR1D1	359	3.028	0.824
PMCR2D1	337	2.810	0.786
PMCR1D9	343	2.944	0.874
PMCR2D9	222	2.348	0.776
CMCR1D1	344	2.790	0.825
CMCR2D1	355	2.861	0.812
CMCR1D6	229	2.742	0.885
CMCR2D6	319	2.331	0.729

### 3.3 Beta diversity

Beta diversity, calculated via Bray-Curtis dissimilarity, revealed shifts in microbial community composition across manure types and culture durations. Principal Coordinates Analysis (PCoA) showed that the first two axes accounted for 36.6% and 32.5% of the total variance, respectively. Initially, poultry manure (PMC) and cow manure (CMC) extracts exhibited similar microbial profiles, with Day 1 samples from both groups clustering closely. However, clear divergence occurred over time. By Day 6 for CMC and Day 9 for PMC, samples formed distinct clusters that were significantly separated from their respective Day 1 counterparts. These results demonstrate a clear separation in microbial communities based on both the manure source and the length of the culture period.

### 3.4 Taxonomic profile of microbiota in copepod culture system

**FIGURE 4:**
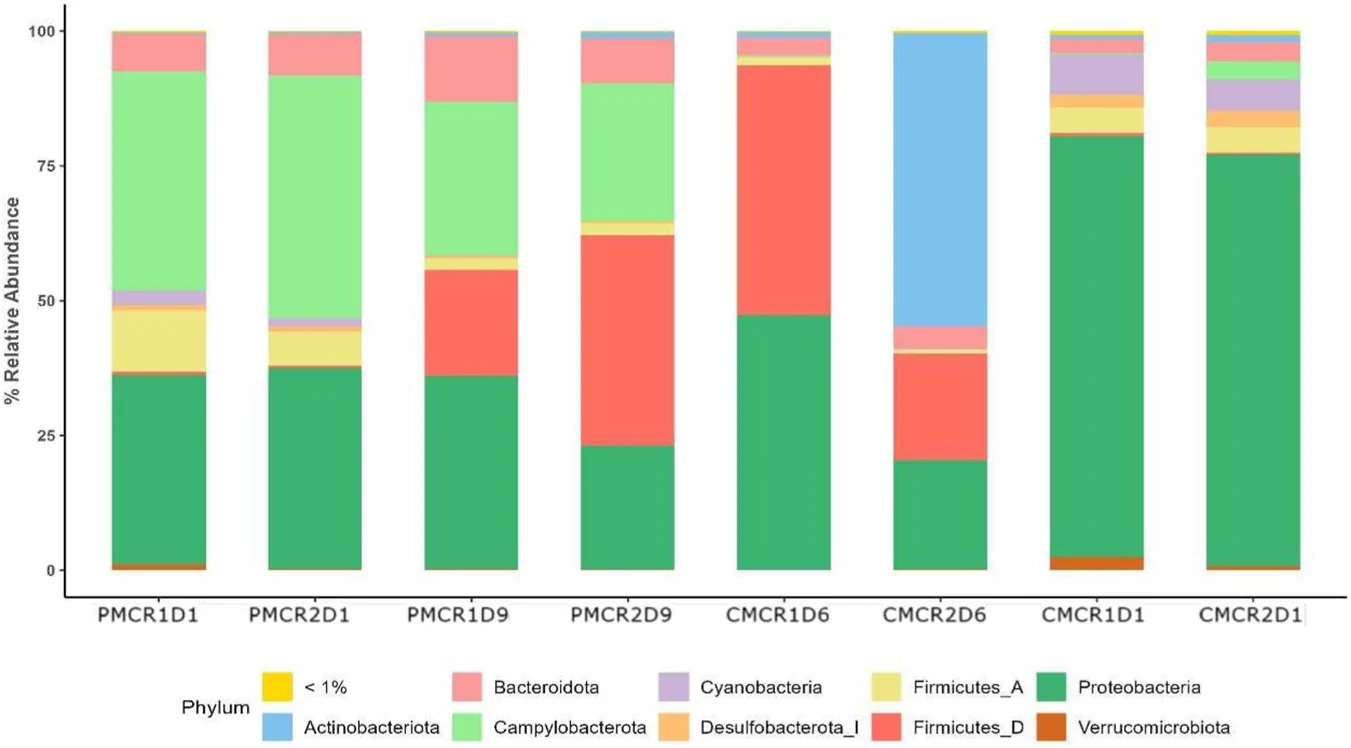
Relative distribution of Phyla at sample level.

**FIGURE 5:**
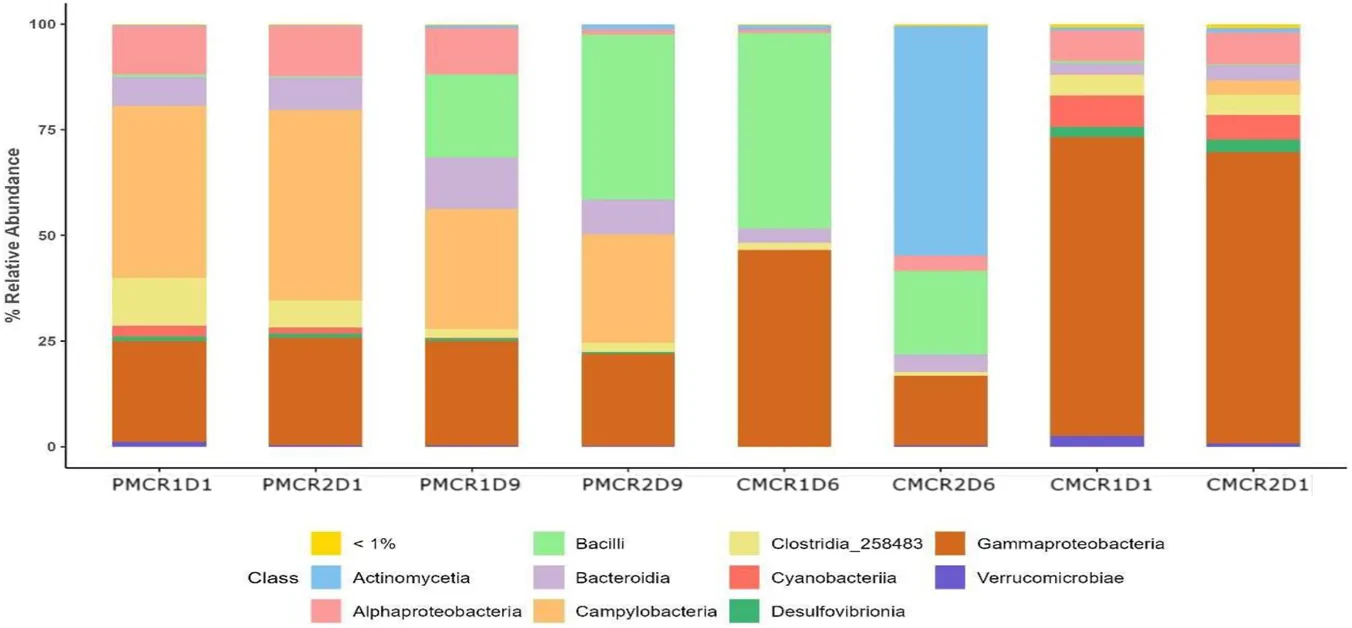
Relative distribution of Class at sample level.

**FIGURE 6:**
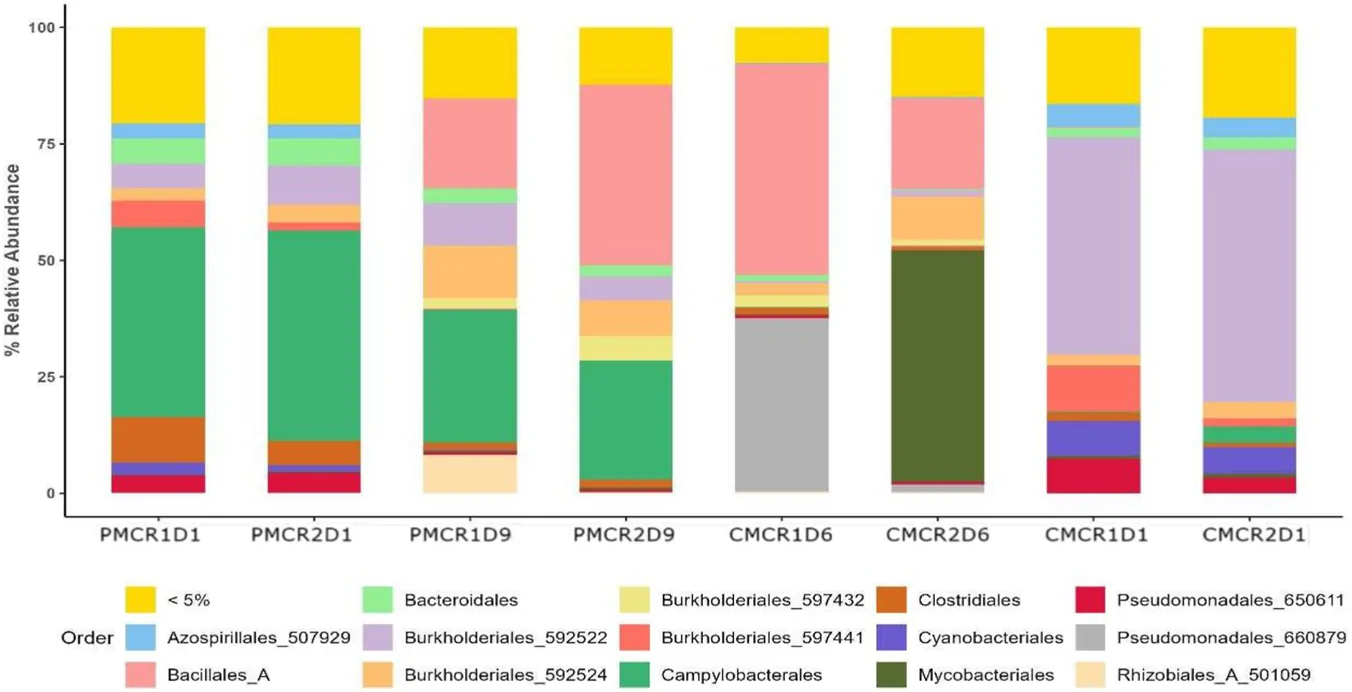
Relative distribution of Order at sample level.

**FIGURE 7:**
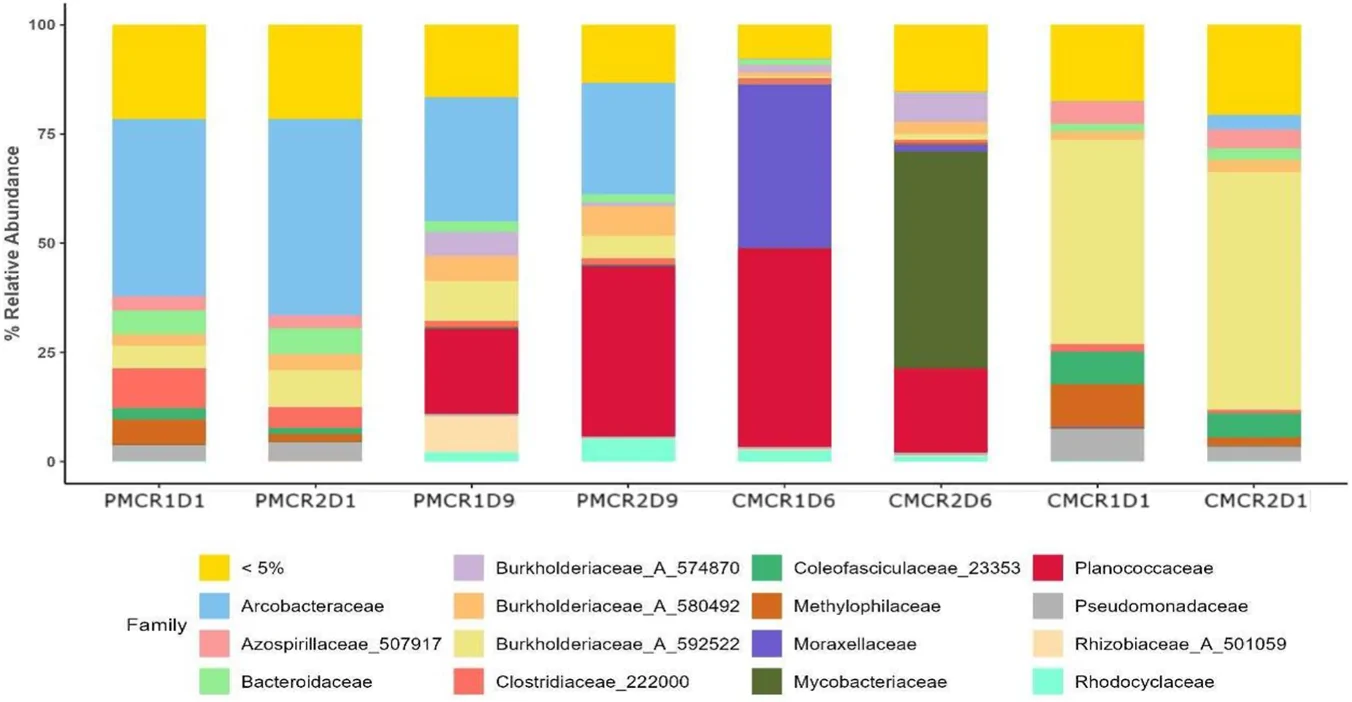
Relative distribution of Family at sample level.

**FIGURE 8:**
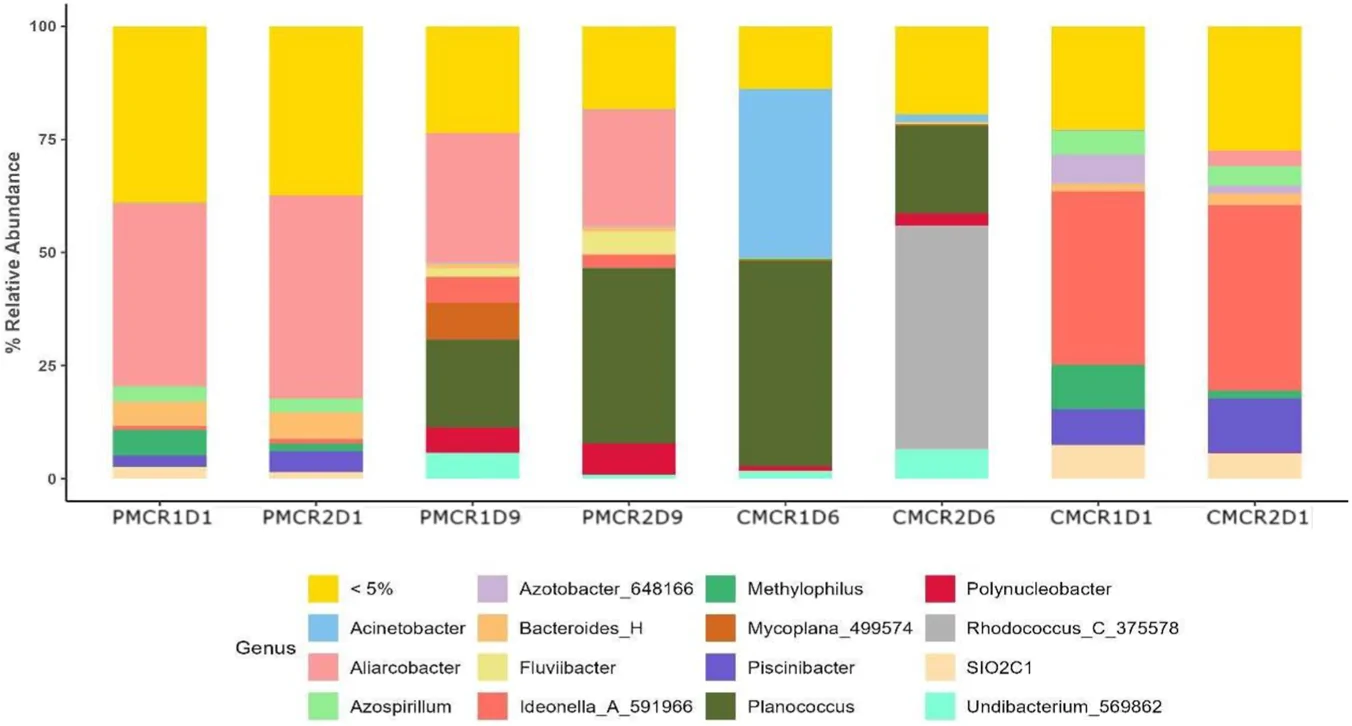
Relative distribution of Genus at sample level.

**FIGURE 9:**
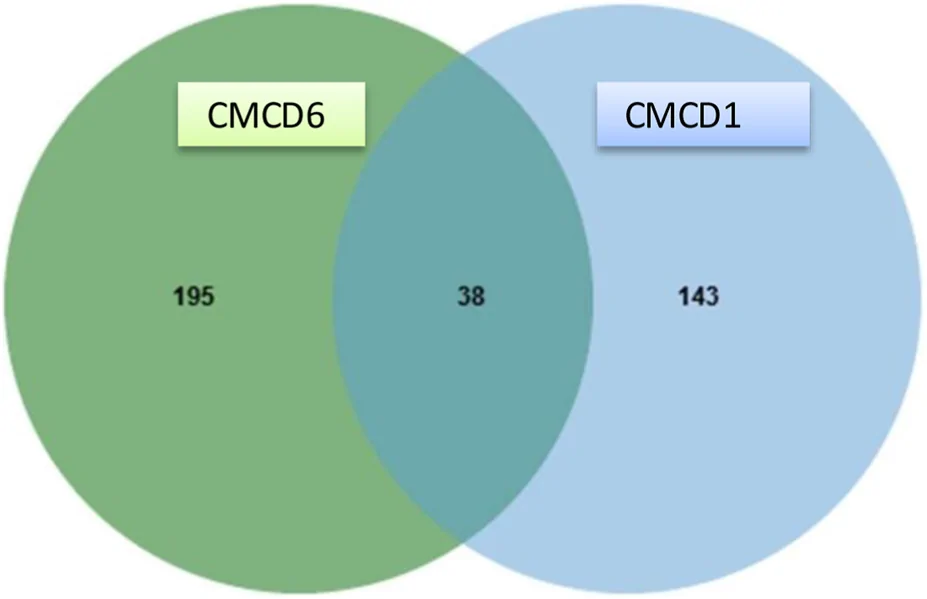
Venn diagram for the comparison of unique ASVs between CMCD6 (Day 6) and CMCD1 (Day 1).

The relative abundance of phylum, class, order, family, genus, and species in each sample is depicted in the figure (Figures 4–9). A suitable cut-off was taken for each classification level to visualize the top abundant taxa. The rare taxa have been collapsed into a separate category, <1% for Phylum and Class and <5% for Family to Species, where the rare taxa in each group with median relative abundance of <1% and <5% respectively, have been collapsed. The plot was generated in R using the Phyloseq package.

As depicted in Figure 4, nine bacterial phyla were detected at relative abundances greater than 1% in both poultry and cow manure extract-based copepod culture systems, including *Actinobacteriota*, *Bacteroidota*, *Campylobacterota*, *Cyanobacteria*, *Desulfobacterota_I*, *Firmicutes_A*, *Firmicutes_D*, *Proteobacteria*, and *Verrucomicrobiota*. In the poultry manure (PMC) extract system, the microbial community on Day 1 was dominated by *Campylobacterota* (41%–45%), *Proteobacteria* (35%–37%), *Firmicutes_A* (6%–11%), and *Bacteroidota* (7%–8%). By Day 9, when the copepod population peaked, the community composition shifted markedly, with *Firmicutes_D* (20%–39%) becoming dominant, followed by *Proteobacteria* (23%–36%), *Campylobacterota* (26%–28%), and *Bacteroidota* (8%–12%). At the class level (Figure 5), *Campylobacteria* was the most abundant on Day 1 (40%–45%), but its relative abundance declined by Day 9. Other dominant classes on Day 1 included *Gammaproteobacteria* (23%–25%), *Alphaproteobacteria* (11%–12%), *Clostridia_258483* (6.3%–11.2%), and *Bacteroidia* (7%–7.9%), whereas by Day 9, the community was primarily composed of *Bacilli* (19.6%–40%), *Campylobacteria* (25.5%–28.4%), and *Gammaproteobacteria* (21.8%–24.7%). Similarly, at the order level (Figure 6), Day 1 samples were dominated by *Campylobacterales* (40.63%–45.09%), *Clostridiales* (5.19%–9.88%), and *Burkholderiales_592522* (5.26%–8.41%), while *Bacillales_A* (19.3%–38.7%), *Campylobacterales* (25.5%–28.4%), and *Burkholderiales_592524* (7.66%–11.24%) were the most abundant orders by Day 9.

In the cow manure (CMC) extract-based copepod culture system, the bacterial community on Day 1 was dominated by *Proteobacteria* (76%–78%), followed by *Cyanobacteria* (6%–7%), *Firmicutes_A* (5%), *Firmicutes_D* (3%–4%), *Verrucomicrobiota* (3%), *Campylobacterota* (3%), and *Desulfobacterota_I* (2%–3%). By Day 6, a significant community shift was observed, characterized by increased abundance of *Actinobacteriota* (54%), along with *Proteobacteria* (20%–47%), *Firmicutes_D* (20%–46%), and *Bacteroidota* (3%–4%) (Figure 4). At the class level (Figure 5), Day 1 samples were dominated by *Gammaproteobacteria* (69%–71%), *Alphaproteobacteria* (7.17%–7.54%), *Cyanobacteriia* (5.74%–7.47%), and *Clostridia_258483* (4.72%–4.75%). By Day 6, the dominant classes included *Gammaproteobacteria* (16.5%–46.4%), *Actinomycetia* (0.19%–54%), *Bacilli* (19.8%–46.3%), and *Bacteroidia* (3.3%–4.1%). Order-level analysis (Figure 6) showed high abundance of *Burkholderiales_592522* (46.7%–54.3%), *Burkholderiales_59744* (1.8%–9.98%), *Cyanobacteriales* (5.74%–7.47%), and *Pseudomonadales_650611* (3.31%–7.41%) on Day 1. On Day 6, dominant orders included *Bacillales_A* (19.4%–45.4%), *Pseudomonadales_660879* (1.64%–37.3%), and *Burkholderiales_592524* (2.6%–9.3%).

### 3.5 Common and unique amplicon sequence variants

**FIGURE 10:**
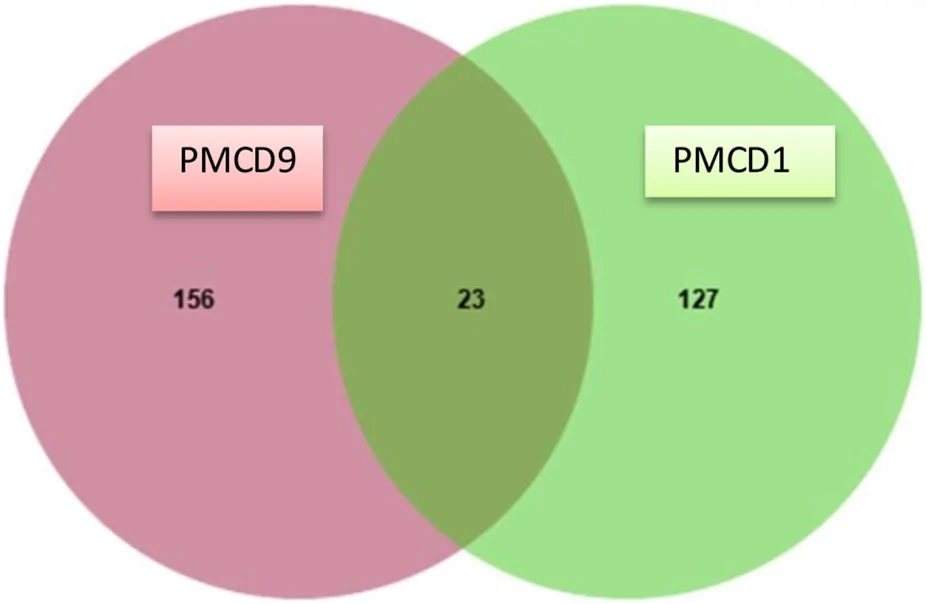
Venn diagram for the comparison of unique ASVs between PMCD9 (Day 9) and PMCD1 (Day 1).

The samples of the group were collapsed together and duplicates were removed, this was then taken as input to create the Venn diagram. The shared and unique Amplicon sequence variants (ASVs) between the groups were plotted as Venn diagram using Jvenn. As illustrated in Figures 9, 10, the number of shared ASVs between CMCD1 (Day 1) and CMCD6 (Day 6)was 38, while PMCD1 (Day 1) and PMCD9 (Day 9) shared 23 ASVs. This indicates a higher ASV overlap in the cow manure extract-based culture system compared to the poultry manure extract-based system.

## 4 Discussion

From both ecological and biotechnological perspectives, characterizing copepod associated bacterial communities is fundamental to optimizing larval rearing systems. Historically, insights into the copepod microbiome were constrained by the limitations of scanning electron microscopy (Nagasawa et al., 1985) and culture dependent techniques (Sochard et al., 1979). While subsequent molecular methods, such as DGGE and PCR cloning, enhanced resolution (Møller et al., 2007), they lacked the depth necessary to resolve the complex multi-trophic interactions within fertilized systems. Traditionally, zooplankton productivity has been attributed to direct nutritional inputs from organic fertilization (Jana and Chakrabarti, 1993; Agbakimi et al., 2017). However, our findings represent a paradigm shift, demonstrating that specific microbial consortia also function as the primary drivers of *M. hyalinus* productivity. By integrating mesocosm scale ecological data with high throughput 16S rRNA sequencing, this study shifts the understanding of organic fertilization toward a microbiome centric framework. Our study aligns with the host-microbe interdependence increasingly recognized in other copepod species (Skovgaard et al., 2015; Vu et al., 2024).

Organic manures, especially poultry manure, have been widely used to enhance zooplankton production due to their rich nutrient content and reported presence of natural hormones that may stimulate productivity (Hakk et al., 2005). Different animal manures vary in steroid content and excretion patterns; cattle release most hormones in faeces, while chickens and pigs excrete them mainly through urine (Hanselman et al., 2003). Unlike phytoplankton, manure derived nutrients are often readily available and more easily assimilated by zooplankton (Khatun et al., 1998). Fish waste contributes essential fatty acids such as EPA and DHA and can support microbial growth (Cho et al., 2014; Ogello et al., 2018). Thus, blends of cow and poultry manure extracts may provide complementary nutrient inputs that support copepod growth, as zooplankton utilize detritus-based carbon sources and associated bacteria linked to improved nutrition and reproduction (Pace et al., 1983; Hagiwara et al., 1994).

In the present study, the combination of cow and poultry manure extracts significantly enhanced the population of *M. hyalinus* compared to individual treatments, consistent with Agbikimi et al. (2017). This improvement is likely associated with the enriched nutrient profile of the combined manure, particularly higher nitrogen and phosphorus levels known to promote zooplankton productivity (Savaş and Guan, 2006; Anitha et al., 2016). The inclusion of rice bran likely contributed additional micronutrients, along with protein (12%–13%) and lipids (16%–20%), supporting its suitability for live feed development (Abbas et al., 2011; Mubarak et al., 2017). Fish waste in the blend likely supplied long chain polyunsaturated fatty acids (EPA and DHA) and supported microbial growth, including taxa with probiotic potential (Cho et al., 2014; Balcázar et al., 2008; Ogello et al., 2018). Together, these components appear to support a detritus-based food web that sustained copepod growth.

The addition of *B. clausii* to the cow manure extract increased copepod density by Day 15, suggesting a delayed probiotic response. Similar trends have been reported in *Tigriopus japonicus* (Sahandi et al., 2023). Probiotics often require time to establish within existing microbial communities and may need continuous application to remain effective (Verschuere et al., 2000). In contrast, no significant effect was observed in the poultry manure treatment, possibly due to established native microbial communities limiting the introduction of external bacteria (Pybus et al., 1994). This indicates that probiotic efficacy depends on substrate type and existing microbiota.

Poultry manure is known to influence microbial communities by altering nutrient levels, pH, and organic matter (Męcik et al., 2023). In this study, declines in Observed ASVs and Shannon index indicate reduced microbial richness and evenness, consistent with previous findings (Męcik et al., 2023). One possible explanation is the presence of residual antibiotics in poultry waste, although this was not measured (Cheng et al., 2019; Tian et al., 2021; Mahdi et al., 2022). The relatively stable Simpson index suggests persistence of dominant, stress-tolerant taxa and a stable core community (Delgado-Baquerizo et al., 2016; 2017; Wagg et al., 2019). While Faissal et al. (2017) reported increased diversity with poultry manure, the present results suggest that selective pressures influenced community composition.

In contrast, cow manure showed only a slight reduction in alpha diversity, indicating relatively stable richness and evenness. This aligns with studies showing that cow manure can enhance microbial diversity and compositional variability (Sayre et al., 2023; Faissal et al., 2017). Its nutrient-rich environment likely supports microbial proliferation and succession (Qaswar et al., 2020; Liu et al., 2021), contributing to functional diversity (Murugan and Kumar, 2013; Tao et al., 2015). Microbial communities in both treatments were initially similar but diverged over time, with distinct profiles by Day 6 (cow manure) and Day 9 (poultry manure). These patterns suggest that both substrate type and incubation duration shape microbial succession, reflecting substrate specific dynamics as reported by Faissal et al. (2017) and influenced by environmental and temporal factors (Hamm et al., 2016; Wang et al., 2017).

The poultry manure system was dominated by *Campylobacterota* and *Proteobacteria*, with *Gammaproteobacteria*, *Alphaproteobacteria*, and *Betaproteobacteria* prevailing. Similar microbiota have been reported in *Acartia tonsa* and *Centropages hamatus* (Skovgaard et al., 2015). These patterns are consistent with observations across marine organisms, where *Proteobacteria, Firmicutes*, and *Bacteroidetes* commonly form core microbiomes shaped by environmental and host factors (Ghanbari et al., 2015; Egerton et al., 2018; Bik et al., 2016; Holt et al., 2021; Forbes et al., 2023). Such taxa are frequently associated with host–microbe interactions across trophic levels.

Initially, the poultry system was dominated by *Aliarcobacter lacus* (40%–45%), a taxon associated with stress prone environments and potential pathogenicity (Müller et al., 2020). By Day 9, its abundance declined (∼25%) as *Planococcus okeanokoites* (16%–39%), reported to exhibit probiotic related traits including antioxidant enzyme activity, became dominant (Li et al., 2021; Zhang et al., 2020). This shift may reflect changes in microbial interactions and nutrient dynamics relevant to copepod growth. *Firmicutes* and *Bacteroidota* are key gut associated phyla linked to carbohydrate degradation and probiotic functions (Wang et al., 2019; Stojanov et al., 2020), and their increased abundance may have influenced nutrient availability and metabolite profiles, although these were not directly measured. *Proteobacteria* remained abundant, with a shift toward *Burkholderiales*, a group involved in nitrogen cycling and organic matter degradation (Dang and Lovell, 2016). The presence of *Burkholderia* spp., known for bioremediation potential (Eissa, 2024), suggests a possible role in maintaining water quality. Overall, microbial succession toward taxa associated with probiotic functions may be linked to observed copepod performance, although causality cannot be confirmed.

In the cow manure system, diverse taxa including *Proteobacteria*, *Cyanobacteria*, *Firmicutes_A Firmicutes_D*, *Actinobacteriota*, and *Bacteroidota* were observed, consistent with previous studies (Skovgaard et al., 2015; Egerton et al., 2018; Forbes et al., 2023). Early dominance by *Ideonella dechloratans* reflects its role in organic matter degradation and micropollutant removal (Spain et al., 2009; Unuofin et al., 2019). By Day 6, increased abundance of *Actinobacteriota* (up to 54%) and *Firmicutes_D* (up to 46%) indicated a shift toward taxa associated with decomposition and nutrient cycling. Reduced *Actinobacteria* abundance has been linked to impaired copepod development (Vu et al., 2024). These groups are known for organic matter degradation, pathogen inhibition, and associations with nutrient assimilation and immune function (Hazarika and Thakur, 2020; Hansen and Bech, 1996; Kakade et al., 2020; Collinder et al., 2003). Their presence, along with *Proteobacteria* and *Bacteroidota,* suggests a stable core microbiome. Increased *Bacteroidota* abundance during peak population aligns with previous findings in *Acartia* sp. (Vu et al., 2024) and reflects roles in carbohydrate and amino acid metabolism (Flint et al., 2012; Liao et al., 2023). Dominant genera such as *Planococcus* and *Acinetobacter* are associated with antioxidant activity, halotolerance, and nutrient cycling (Li et al., 2021; Zhang et al., 2020), which may contribute to culture stability.

Our findings report that the divergence in copepod growth patterns between treatments can be linked to the ‘microbial succession’ triggered by the manure types. Cow manure based combinations (CMC) promoted a rapid bloom of *Actinobacteriota* and *Firmicutes*; these taxa are recognized as efficient decomposers of complex organic matter, facilitating rapid nutrient mineralization (Hazarika and Thakur, 2020; Kakade et al., 2020). This facilitated a fast nutrient release and an early peak in copepod density by Day 6. Conversely, poultry manure (PMC) induced a more gradual successional shift, characterized by a transition from potential pathogens (*Aliarcobacter*) to beneficial taxa such as *Planococcus* (Li et al., 2021; Zhang et al., 2020). This microbial transition explains the delayed but sustained population build up observed in the poultry based systems, suggesting that while cow manure serves as an immediate growth ‘booster,’ poultry manure supports long term population stability through the establishment of a specialized, probiotic like bacterial consortium.

The higher number of shared ASVs in the cow manure system indicates greater microbial stability over time. This may relate to more consistent nutrient composition and potentially lower antimicrobial residues compared to poultry manure. Poultry manure is known to contain residual antibiotics and heavy metals (Zhou et al., 2020; Karcı and Balcıoglu, 2009), which can impose selective pressures and alter microbial structure (Męcik et al., 2023).

## 5 Conclusion

The study highlights the importance of mesocosm based approach combined with16S rRNA tool to provide to insights into fertilization strategies for optimizing sustainable copepod based live feed production in freshwater aquaculture. The present study demonstrated that the composition of organic manure filtrates has a significant influence on the growth performance of *M. hyalinus*. Cow manure based media combination (CMC) promoted growth rapidly during initial days of culture, while poultry manure based media (PMC) supported sustained growth. The associated microbiota in the *M. hyalinus* culture system revealed that distinct microbiota were associated with high population densities in cow manure and poultry manure based culture system. The identified microbes through 16S rRNA sequencing provide a basis for formulating targeted bacterial consortia as a bioinoculant product for the *M. hyalinus* culture system. These findings also highlight the critical role of manure composition and microbial community structure in optimizing copepod culture systems for aquaculture, offering a low cost strategy for enhancing live feed productivity. Future research should focus on the selective isolation and culturing of the dominant bacterial consortia identified in our study. Developing these as standardized bioinoculants could revolutionize copepod mass production by ensuring a predictable and beneficial microbiome, reducing the industry’s reliance on raw organic waste and improving biosecurity in freshwater aquaculture systems.

## Data Availability

The original contributions presented in the study are publicly available. This data can be found in the NCBI repository with the Bio project accession number: PRJNA1507228, at: https://www.ncbi.nlm.nih.gov/sra/PRJNA1507228.
