# Supplementary material for: Microbial metacommunity dynamics associated with the cyclopoid copepod Mesocyclops hyalinus (Rehberg, 1880) in freshwater mesocosm using low-cost organic media
Source: Front Syst Biol. 2026 Aug 11;6:1845217. doi: 10.3389/fsysb.2026.1845217 (PMC13503331; doi:10.3389/fsysb.2026.1845217)
Supplement: Supplementary file 1 [file DataSheet1.docx]

**SUPPLEMENTARY FILE**

**Gel Image**

1. **Agarose gel profile of DNA co-electrophoresed with 1KB DNA Ladder (L)**

2. **Tape station profile of library generated where EL denotes electronic ladder**
